# Malignant Triton Tumor of the Ileocolic Mesentery with Neurofibromatosis Type 1: A Case Report and Literature Review

**DOI:** 10.70352/scrj.cr.25-0096

**Published:** 2025-06-10

**Authors:** Kenichi Ishibayashi, Hirotaka Kitamura, Sho Tsuyama, Akane Yoshikawa, Maika Zasu, Yusuke Ikku, Tetsuya Asakawa, Katsuya Gunjikake, Yosuke Kito, Takahisa Yamaguchi, Yoshinao Ohbatake, Shiro Terai, Kazuyoshi Katayanagi, Shinichi Kadoya, Hiroshi Minato, Hiroyuki Bando

**Affiliations:** 1 Department of Gastroenterological Surgery, Ishikawa Prefectural Central Hospital, Kanazawa, Ishikawa, Japan; 2 Department of Diagnostic Pathology, Ishikawa Prefectural Central Hospital, Kanazawa, Ishikawa, Japan; 3 Department of Medical Oncology, Ishikawa Prefectural Central Hospital, Kanazawa, Ishikawa, Japan

**Keywords:** malignant triton tumor, mesentery, neurofibromatosis type 1, abdominal tumor, biopsy, malignant peripheral nerve sheath tumor

## Abstract

**INTRODUCTION:**

Malignant triton tumors (MTTs) are rare tumors histologically defined as malignant peripheral nerve sheath tumors with additional rhabdomyoblastic differentiation. MTTs occur more frequently in patients with neurofibromatosis type 1 (NF1). MTTs are most commonly found in the extremities, head and neck, and trunk; however, no cases in the mesentery have been reported. In this report, we describe a case of primary MTT of the mesentery in a patient with NF1.

**CASE PRESENTATION:**

A 29-year-old woman with NF1 visited a clinic for abdominal pain and was referred to our hospital for treatment of an abdominal tumor detected by CT. Contrast-enhanced CT showed a 20-cm irregular mass on the right side of the abdominal cavity. The mass was extensively bordered by the superior mesenteric vein (SMV), but there was no obvious distant metastasis. The patient was diagnosed with a mesenteric tumor with SMV invasion. An open biopsy was performed, and the histopathological diagnosis was MTT. The patient was discharged on the 11th day after surgery but returned to our hospital on the 17th day due to abdominal pain. The tumor had markedly enlarged on CT, and a semi-emergent tumor resection was performed. An enlarged mass was detected, but no peritoneal dissemination was observed. The tumor was in extensive contact with the SMV, but it could be dissected. A right hemicolectomy was performed, and the tumor was removed. Histopathological findings revealed a 26.5 × 17.5 × 9.5 cm tumor in the ileocolic mesentery. The dissected surface was negative for margins, with no exposed tumors. The histopathological diagnosis was MTT. Follow-up CT on postoperative day 14 revealed multiple peritoneal nodules. The patient was diagnosed with recurrent peritoneal dissemination and treated with two courses of doxorubicin and one course of pazopanib. However, she died 5 months postoperatively due to worsening peritonitis carcinomatosis.

**CONCLUSIONS:**

We present a case of primary MTT of the mesentery. Our findings suggest that biopsy may lead to peritoneal dissemination. In patients with suspected MTT, such as those with NF1, abdominal cavity biopsy should be avoided, and diagnostic treatment should prioritize surgical resection.

## Abbreviations

GIST
: gastrointestinal tumor
MPNST
: malignant peripheral nerve sheath tumor
MTT
: malignant triton tumor
NF1
: neurofibromatosis type 1
SMV
: superior mesenteric vein

## INTRODUCTION

A malignant triton tumor (MTT) is a rare subtype of malignant peripheral nerve sheath tumor (MPNST). Histologically, MTTs are characterized by MPNST features combined with rhabdomyoblastic differentiation, a critical criterion for diagnosis. MTT occurs more frequently in patients with neurofibromatosis type 1 (NF1), with 44%–69% of MTT patients having NF1.^1)^ MTT is an extremely aggressive tumor with a 5-year survival rate of 26%, a metastatic rate of 48%, and a local recurrence rate of 43%.^2–4)^ While MTTs are most frequently identified in the extremities, head, neck, and trunk, no cases involving the mesentery have been documented to date. Here, we describe a case of primary mesenteric MTT in a patient with NF1. Documenting such rare cases is crucial for enhancing the understanding of diagnostic approaches and therapeutic strategies for these aggressive and understudied tumors.

## CASE PRESENTATION

**Fig. 1:**
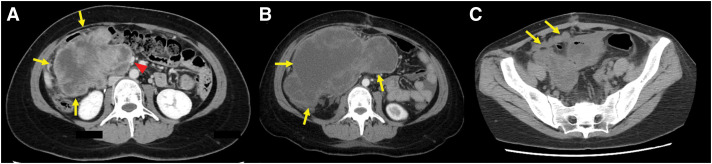
(**A**) Contrast-enhanced computed tomography indicated a large, irregular mass (arrows) on the right side of the abdominal cavity. The mass was extensively bordered by the narrow superior mesenteric vein (arrowhead). (**B**) Contrast-enhanced computed tomography revealed the markedly enlarged tumor (arrows). (**C**) A follow-up CT on postoperative day 14 revealed multiple peritoneal nodules (arrows).

A 29-year-old woman visited a previous clinic for abdominal pain and was referred to our hospital for treatment of a 20-cm abdominal tumor detected on CT. She had a history of NF1, and her family history was unremarkable. Upon physical examination, an adult-sized head mass was palpated in the right upper abdomen. Bilateral leg edema and numerous café-au-lait spots were observed on the skin. Laboratory tests, including tumor marker analysis, revealed no abnormalities. Contrast-enhanced CT indicated a large, irregular mass on the right side of the abdominal cavity, which was thought to be primarily located in the ascending mesocolon (**Fig. 1A**).

**Fig. 2:**
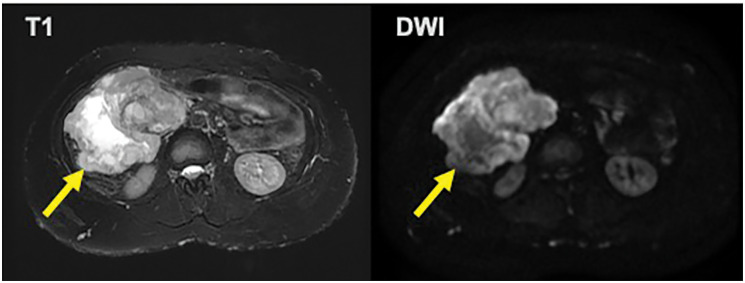
The tumor (arrow) showed high signal intensity on T2 and diffusion-weighted magnetic resonance imaging.

**Fig. 3:**
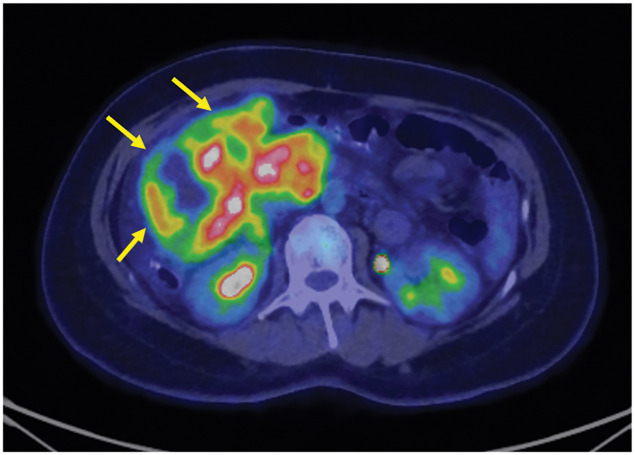
Positron emission tomography-computed tomography showed an irregular accumulation of fluorodeoxyglucose in the tumor (arrows).

The mass was extensively bordered by the narrow superior mesenteric vein (SMV), which was suspected to be invaded. Ascites was present, but no obvious distant metastasis was observed. The tumor showed high signal intensity on T2 and diffusion-weighted magnetic resonance imaging (**Fig. 2**). Positron emission tomography-CT showed an irregular accumulation of fluorodeoxyglucose in the tumor (**Fig. 3**). The patient was diagnosed with a mesenteric tumor with SMV invasion, and an open biopsy was performed. Through a 12-cm midline incision, a 20-cm mass was identified in the right abdominal cavity, attached to the mesentery. A 1-cm protruding portion was resected using an electric scalpel, and a specimen was obtained. The excised area was coagulated, and hemostasis was achieved with soft coagulation.

Histopathological findings of the biopsy specimen showed spindle-shaped atypical cells proliferating in bundles, accompanied by increased mitotic activity. Immunohistochemical staining was negative for S100, with a high Ki-67 positivity rate. Additionally, some cells exhibited eosinophilic cytoplasm and were positive for desmin and myogenin, indicative of rhabdomyoblastic differentiation. These findings led to a diagnosis of MTT.

The patient was discharged on the 11th day after surgery but returned on the 17th day due to abdominal pain. As the tumor had markedly enlarged on CT, we decided to perform semi-emergent tumor resection (**Fig. 1B**).

Through a median incision, a 26-cm tumor was identified in the right abdominal cavity. Minimal ascites were present, and no peritoneal dissemination was observed. Dissection revealed the tumor’s location in the ileocecal mesentery, with no retroperitoneal or duodenal invasion. The tumor was in extensive contact with the SMV but could be dissected. A right hemicolectomy was performed, and the tumor was removed. The operation time was 4 hours and 34 minutes, and the blood loss was 1260 mL.

**Fig. 4:**
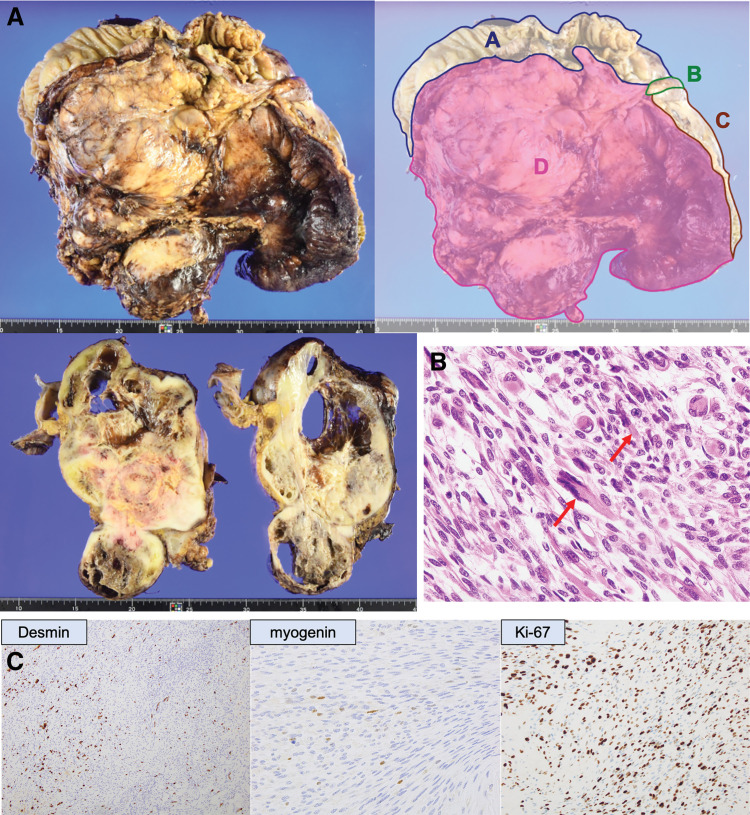
(**A**) Histopathological findings revealed a 26.5 × 17.5 × 9.5 cm tumor in the ileocolic mesentery. (**A**) Ascending colon, (**B**) appendix, (**C**) ileum, (**D**) tumor. (**B**) Microscopically, atypical spindle-shaped cells proliferated in bundles, alternating between high- and low-cellular regions, with edematous or myxoid stroma. Acidophilic rhabdomyoblastic cells (arrows) were observed sporadically in some areas. (**C**) Immunohistochemistry demonstrated positive staining for myogenin and desmin in the rhabdomyoblastic cells. The Ki-67 positivity rate was notably elevated, with 79.2% of the cells being Ki-67 positive.

Histopathological findings revealed a 26.5 × 17.5 × 9.5 cm tumor in the ileocolic mesentery, with some direct invasion into the muscularis propria of the colon (**Fig. 4A**). The dissected surface was negative for margins, with no exposed tumor. Microscopically, atypical spindle-shaped cells proliferated in bundles, alternating between high- and low-cellular regions, with edematous or myxoid stroma. Acidophilic rhabdomyoblastic cells were observed sporadically in some areas (**Fig. 4B**). The tumors exhibited increased mitosis and extensive necrosis. Immunohistochemistry demonstrated positive staining for epithelial membrane antigen, myogenin, and desmin in the rhabdomyoblastic cells. The Ki-67 positivity rate was notably elevated, with 79.2% of the cells being Ki-67 positive (**Fig. 4C**). Based on these findings, a diagnosis of MTT was confirmed.

A follow-up CT on postoperative day 14 revealed multiple peritoneal nodules (**Fig. 1C**). She was diagnosed with recurrent peritoneal dissemination and treated with two courses of doxorubicin and one course of pazopanib, but died 5 months postoperatively due to worsening peritonitis carcinomatosis.

## DISCUSSION

Two critical clinical considerations were identified: first, MTT can occur in the mesentery, and second, a biopsy of MTT may lead to peritoneal dissemination.

Initially, MTT was observed in the mesentery. MTT is a rare tumor, and the frequency of its occurrence varies according to reports, but it is most commonly reported in the extremities, head and neck, and trunk.^3,5)^ Abdominal MTT is rare, with one case described as a duodenal primary tumor^6)^ and eight cases as retroperitoneal primary tumors.^7–13)^ However, mesenteric MTT has not been previously reported. The 10 reported cases of abdominal tumors, including our case, are summarized in **Table 1**. Most patients presented with abdominal pain and exhibited abdominal masses with an average size of 13.8 cm. Patients with duodenal tumors exhibited symptoms of anemia, whereas those without a primary gastrointestinal tumor were difficult to detect until the tumor enlarged and abdominal pain developed.

**Table 1:** The 10 reported cases of abdominal MTT

Referrence	Year	Author	Age	Sex	Chief complaint	Location	Tumor size (cm)	Surgical resection	Chemotherapy	Radiation	Outcomes
7	2009	Hoshimoto S	21	M	Left abdominal mass	Retroperitoneum	19	Incomplete	+	–	Lung metastasis, DOD 14 months after surgery
8	2011	Nirhale D	62	M	Abdominal mass	Retroperitoneum	10.5	Complete	–	–	Disease-free 1 years
9	2012	Li Z	32	M	Epigastric pain	Retroperitoneum	16	Complete	–	–	Liver metastasis, DOD 2.5 months after surgery
10	2013	Mijovic Z	57	F	Abdominal pain	Retroperitoneum	7.5	Complete	–	+	Disease-free 8 months
6	2018	Asahi Y	64	F	Chest oppression	Duodenum	5.5	Complete	–	–	Disease-free 8.5 years
11	2019	Bian Y	13	F	Weight loss	Retroperitoneum	NA	Complete	+	+	Recurrence at thoracic vertebra, DOD
11	2019	Bian Y	57	F	Right abdominal mass	Retroperitoneum	NA	Complete	–	+	Local recurrence, alive with disease
12	2020	Hou Z	0 (8 month)	F	Abdominal mass	Retroperitoneum	11	Complete	–	–	Local recurrence and peritoneal dissemination, DOD 3 months after surgery
13	2022	Liu M	21	M	Abdominal pain, weight loss	Retroperitoneum	21	Complete	–	–	Local recurrence and peritoneal dissemination, DOD 2 months after surgery
Present case	2024	Ishibayashi K	29	F	Abdominal pain	Ileocolic mesentery	20	Complete	+	–	Peritoneal dissemination, DOD 5 months after surgery

MTT, malignant triton tumor; DOD, dead of diseases

Second, a biopsy of MTT may lead to peritoneal dissemination; therefore, careful consideration is required when formulating a treatment plan for abdominal tumors in which MTT is suspected. In reports of MTT occurring in the extremities, head and neck, or trunk, treatment was often initiated after a pathological diagnosis was confirmed by biopsy, and no adverse events, such as biopsy-related metastasis, have been reported. Additionally, only one case has been reported in which a CT-guided needle biopsy was performed to diagnose a primary MTT of the posterior mediastinum, and no metastasis was observed following the biopsy.^14)^ In the nine reported cases of abdominal tumors, excluding the duodenal primary case, all were diagnosed without biopsy and treated with tumor resection as a diagnostic procedure.^7–13)^ Asahi et al. reported that in the duodenal primary case, the tumor was exposed within the intestinal lumen, allowing endoscopic biopsy; however, no adverse events occurred following the procedure.^6)^ In our case, tumor resection was performed following an open biopsy; however, peritoneal dissemination recurred in the early postoperative period. It cannot be ruled out that the open biopsy contributed to the dissemination. Although there are no reports of needle biopsy for MTT presenting as an abdominal tumor, it is considered that performing the procedure via the abdominal cavity carries a risk of peritoneal dissemination, similar to that associated with open biopsy. In our case, since the infiltration of the SMV by the tumor could not be ruled out, we first performed an open biopsy, but ultimately, the tumor was resectable. At the time of exploratory laparotomy, it should have been thoroughly evaluated whether resection was feasible, and if so, tumor resection should have been performed as diagnostic treatment at that time. If resection was deemed impossible at the time of exploratory laparotomy, a biopsy should have been performed, followed by chemotherapy or radiotherapy. As MTT can cause peritoneal dissemination during biopsy, diagnostic and treatment strategies should be approached with caution. In cases where MTT cannot be ruled out, we recommend avoiding biopsies via the abdominal cavity.

MTT occurs more frequently in patients with NF1, with 44%–69% of MTT patients having NF1.^1)^ Compared with sporadic cases, MTT associated with NF-1 tends to present at a younger age, occurs more commonly in males, and is associated with a poorer prognosis.^15)^ NF1 is an autosomal dominant genetic disorder affecting approximately 1 in 3000 individuals, and it predisposes patients to a wide range of neoplasms, including MTT.^16)^ The overall risk of tumor development in patients with NF1 is estimated to be 5%–15% higher than in the general population.^17)^ The underlying mechanism involves mutations in the NF1 gene, which encodes neurofibromin, a negative regulator of the RAS oncogenic pathway.^18–20)^ Inactivating mutations in the NF1 gene lead to downstream activation of the mitogen-activated protein kinase, phosphatidylinositol 3-kinase/protein kinase B, and mechanistic target of rapamycin signaling. This dysregulation results in uncontrolled cellular growth, differentiation, and survival, contributing to the pathogenesis of NF1 and NF1-associated tumors. The most frequently reported malignant tumors in patients with NF1 and their respective frequencies are as follows: low-grade glioma (16.6%), MPNST (15.1%), breast cancer (2.9%), high-grade glioma (1.7%), pheochromocytoma (1.2%), gastrointestinal stromal tumors (GIST) (1.2%), and melanoma (0.9%).^21)^ Focusing specifically on abdominal tumors, GIST is the most common, followed by MPNST.^22)^

GIST, MPNST, and MTT associated with NF1 commonly exhibit poor responses to chemotherapy or radiotherapy, making complete surgical resection the standard treatment. NF1-associated GISTs are caused by hyperplasia of Cajal cells due to NF1 mutations. Immunostaining for KIT was positive, but c-kit and PDGFRA mutations were absent. Therefore, sporadic and familial GIST can be treated with tyrosine kinase inhibitors, but these inhibitors are ineffective for NF1-associated GIST.^22)^ Lymph node dissection is not indicated for GIST or MPNST. Although there is no established policy regarding the role of lymph node dissection in MTT, as a subtype of MPNST, it is considered unnecessary. Therefore, when the histological diagnosis of abdominal tumors in NF1 patients is not available, a treatment strategy based on surgery should be established, assuming that chemoradiotherapy is not effective.

Chemotherapy for MTT is currently not well established. However, for MPNST, chemotherapy similar to that for advanced soft tissue sarcomas is recommended. Since MTT is a type of MPNST, similar chemotherapy regimens are considered appropriate. Specifically, the standard first-line treatment consists of doxorubicin-based chemotherapy, either as monotherapy or in combination with ifosfamide.^23)^ There is no established standard for second-line therapy, and available options include trabectedin, eribulin, and pazopanib. However, no consensus exists regarding the optimal regimen.^24,25)^ In the present case, two cycles of doxorubicin were administered as first-line therapy, but the disease continued to progress. Due to severe nausea at that time, pazopanib, which is associated with a lower incidence of gastrointestinal side effects, was selected as second-line therapy; however, the therapeutic response was limited.

## CONCLUSIONS

This study reports a rare case of MTT occurring in the mesentery and identifies that biopsy may lead to peritoneal dissemination. In patients with suspected MTT, such as those with NF1, abdominal cavity biopsy should be avoided, and diagnostic treatment should prioritize surgical resection.

## ACKNOWLEDGMENTS

The authors would like to thank Editage (www.editage.com) for the English language editing.

## DECLARATIONS

### Funding

This study did not receive any specific grants from funding agencies in the public, commercial, or not-for-profit sectors.

### Author’s contributions

KI designed the study and prepared the manuscript.

HK performed the surgery.

YO and YK treated the patient.

MZ, TA, KK, KG, YI, TY, AY, ST, YK, and HM provided the previous case reports.

SK and HB supervised the study.

All the authors have read and approved the final version of the manuscript.

### Availability of data and materials

Not applicable.

### Ethics approval and consent to participate

Patient privacy was considered and the manuscript did not include any identifying information.

The patient has consented to participate in the study. Our institution does not require ethical approval for the publication of case reports.

### Consent for publication

The patient provided informed consent for the publication of this case.

### Competing interests

The authors declare that they have no competing interests.
